# Association of left ventricular abnormalities with incident cerebrovascular events and sources of thromboembolism in patients with chronic Chagas cardiomyopathy

**DOI:** 10.1186/s12968-022-00885-x

**Published:** 2022-11-03

**Authors:** Henrique Turin Moreira, Gustavo Jardim Volpe, Gustavo Marques Mesquita, Maria Fernanda Braggion-Santos, Antonio Pazin-Filho, José Antonio Marin-Neto, André Schmidt

**Affiliations:** Division of Cardiology, Department of Internal Medicine, Ribeirão Preto Medical School, Hospital das Clínicas de Ribeirão Preto, University of São Paulo, Avenida dos Bandeirantes, 3900, Ribeirão Preto, SP 14048-900 Brazil

**Keywords:** Chagas disease, Left ventricular dysfunction, Magnetic resonance imaging, Stroke, Transient ischemic attack, Atrial fibrillation

## Abstract

**Background:**

Although Chagas cardiomyopathy is related to thromboembolic stroke, data on risk factors for cerebrovascular events in Chagas disease is limited. Thus, we assessed the relationship between left ventricular (LV) impairment and cerebrovascular events and sources of thromboembolism in patients with Chagas cardiomyopathy.

**Methods:**

This retrospective cohort included patients with chronic Chagas cardiomyopathy who underwent cardiovascular magnetic resonance (CMR). CMR was performed with a 1.5 T scanner to provide LV volumes, mass, ejection fraction (LVEF), and myocardial fibrosis. The primary outcome was a composite of incident ischemic cerebrovascular events (stroke or transient ischemic attack—TIA) and potential thromboembolic sources (atrial fibrillation (AF), atrial flutter, or intracavitary thrombus) during the follow-up.

**Results:**

A total of 113 patients were included. Median age was 56 years (IQR: 45–67), and 58 (51%) were women. The median LVEF was 53% (IQR: 41–62). LV aneurysms and LV fibrosis were present in 38 (34%) and 76 (67%) individuals, respectively. The median follow-up time was 6.9 years, with 29 events: 11 cerebrovascular events, 16 had AF or atrial flutter, and two had LV apical thrombosis. In the multivariable model, only lower LVEF remained significantly associated with the outcomes (HR: 0.96, 95% CI: 0.93–0.99). Patients with reduced LVEF lower than 40% had a much higher risk of cerebrovascular events and thromboembolic sources (HR: 3.16 95% CI: 1.38–7.25) than those with normal LVEF. The combined incidence rate of the combined events in chronic Chagas cardiomyopathy patients with reduced LVEF was 13.9 new cases per 100 persons-year.

**Conclusions:**

LV systolic dysfunction is an independent predictor of adverse cerebrovascular events and potential sources of thromboembolism in patients with chronic Chagas cardiomyopathy.

**Supplementary Information:**

The online version contains supplementary material available at 10.1186/s12968-022-00885-x.

## Background

Chagas disease is caused by infection with the protozoan parasite *Trypanosoma cruzi (T. cruzi),* transmitted to human beings primarily by contact with blood-sucking triatomine bugs. However, other transmission routes are still possible, such as blood transfusion, solid organ transplantation, congenitally from mother to fetus, and oral infection through ingestion of contaminated food or drink [1]. Despite the substantial reduction of acute cases of Chagas disease by transfusions and direct vectorial transmission in the last decades, the World Health Organization recently estimated 6–7 million people infected by *T. cruzi* worldwide, while 75 million people are at risk of infection [2]. Furthermore, population migratory moves have been related to increasing cases detected in non-endemic areas, mainly in the United States and European countries [3, 4].

Chronic Chagas cardiomyopathy, the most severe and frequent manifestation of Chagas disease chronic phase, may lead to heart failure, arrhythmias, and systemic and pulmonary thromboembolic events [5]. Even though Chagas cardiomyopathy is independently related to ischemic stroke, cerebrovascular events may be an unrecognized and neglected adverse outcome in most patients with Chagas disease [6, 7]. Cardioembolism is the leading cause of stroke in this clinical scenario, especially in the presence of atrial fibrillation (AF) [8]. However, other cardiac abnormalities may also play a role, including left ventricular (LV) systolic dysfunction, apical aneurysm, and mural thrombus [9, 10]. It is noteworthy that stroke can be the first sign of Chagas disease in asymptomatic patients with this condition, irrespective of detection of LV dysfunction or supraventricular arrhythmia [11].

However, data about the association of LV impairment with cerebrovascular events in patients with Chagas disease is limited, so a debate continues about the best strategy for preventing and managing stroke and transient ischemic attack (TIA) in this clinical setting [12]. Despite this uncertainty, it is intuitive that identifying risk factors for these adverse outcomes in such patients, especially using a noninvasive method of reference for cardiovascular imaging, is crucial for a thorough strategy to prevent these ominous events.

Thus, the purpose of this study was to investigate the relationship between LV morphological and functional parameters, assessed by cardiovascular magnetic resonance (CMR), with the incidence of ischemic cerebrovascular events and potential sources of thromboembolism in patients with chronic Chagas cardiomyopathy.

## Methods

### Study participants

In this time-to-event analysis, we retrospectively collected data from 140 adult patients aged ≥ 18 years and with chronic Chagas cardiomyopathy who underwent CMR from October 2009 to December 2013. All patients were followed in the outpatient clinics at the Hospital das Clínicas de Ribeirão Preto, University of São Paulo, Brazil. Chagas disease was defined by the positivity of at least two different serological tests: enzyme-linked immunosorbent assay, indirect immunofluorescence, or hemagglutination. CMR was requested according to a prior study protocol from our group, published elsewhere [13]. All patients had at least one marker of Chagas cardiomyopathy, defined as the presence of LV global or regional dysfunction or LV scar on CMR**.** Exclusion criteria were evidence of other types of cardiomyopathies, known obstructive epicardial coronary artery disease, primary valvular heart disease, or contraindication for CMR, including cardiac implantable electronic devices and claustrophobia, as previously described [13]. Additional exclusion criteria were previous stroke (n = 3), AF (n = 12), or oral anticoagulant use at the time of CMR (n = 11). One patient was excluded from the study due to loss of follow-up immediately after CMR (n = 1). A total of 113 patients were ultimately included in the study. The study protocol was approved by the institutional research ethics committee and was conducted following the Helsinki Declaration (process number 4913/2010). All participants gave written informed consent to the study.

### Cardiac magnetic resonance

Images were acquired using a 1.5 T scanner (Achieva, Philips Healthcare, Best, The Netherlands), with a 5-element coil SENSE. Cine imaging was obtained by balanced steady-state–free precession pulse sequence electrocardiographic (ECG) gated, with a ventricular short-axis slice thickness of 8 mm and a gap of 2 mm between slices. Myocardial late gadolinium enhancement (LGE) evaluation was assessed using a T1-weighted inversion recovery fast gradient echo pulse sequence, applied 10 min after intravenous infusion of 0.2 mmol of gadolinium-based contrast (Omniscan; General Electric Healthcare, Chicago, Illinois, USA), with ventricular slices thickness of 10 mm and no gap between them. Detailed CMR protocol parameters are reported in Additional file 1: Table S1. Exams were stored in the DICOM pattern and evaluated by a single experienced reader, blinded to patients' data, using MASS software (Leiden University, Leiden, The Netherlands). LV ejection fraction (LVEF) was examined in cine images by the Simpson disk summation technique. The first basal slice was defined as the one immediately adjacent to the atrioventricular junction. End-diastolic and end-systolic volumes were determined as the largest and the smallest volumes during the cardiac cycle, respectively, for endocardial manual delineation. Papillary muscles and trabeculae were considered part of the ventricular cavity. Myocardial scar areas were quantified using the full-width at half-maximum criterion, which uses half the maximal signal intensity of the enhanced area as the threshold to define fibrosis, as previously described [13]. LV fibrosis mass was calculated as the product of LGE volume and density of myocardial tissue (1.05 g/mL).

### Clinical assessment and follow-up

The study follow-up time was defined from the CMR examination to the last clinical visit until December 31st, 2020. Demographics and clinical data were obtained when the participants underwent the CMR. All participants were followed in a specialized clinic for Chagas disease, with visits performed at least once a year but more frequently if clinically necessary. Clinical management was performed according to the Latin American Guidelines for the Diagnosis and Treatment of Chagas Heart Disease [14]. As a routine protocol, patients underwent ECG and 24-h Holter monitoring if there was a change in clinical status, heart palpitations, or arrhythmias detected by physical examination.

The study endpoint was a composite of incident ischemic cerebrovascular events (stroke or TIA) and potential thromboembolic sources (AF, atrial flutter, or intracavitary thrombus). According to a hierarchical order, only one outcome was computed for two or more overlapping incident events, beginning with stroke or TIA, followed by intracavitary thrombus and AF or flutter. Cerebrovascular events were identified from medical records reviewed by two physician members of the study group, with a third senior physician to resolve disagreements, if necessary. Diagnosis of stroke was determined as a focal neurologic deficit of sudden onset, persisting for more than 24 h, without a known alternative to a vascular cause, and confirmed by clinical examination or radiological findings according to Stroke Data Bank and World Health Organization criteria [15, 16]. TIA was defined as focal neurological symptoms lasting less than 24 h. Clinical characteristics at the time of the cerebrovascular event were also retrieved from the medical reports, including neurologic manifestations, cranial computed tomography scan, ECG, echocardiography, and imaging of intra- and extracranial arteries. AF was defined by irregular R–R intervals, absence of distinct P waves, and signs of irregular atrial activations on ECG [17]. Atrial flutter was characterized by an organized atrial tachycardia with a rate of ≥ 240 beats/min lacking an isoelectric baseline between deflections [18]. Intracavitary thrombus was determined from invasive or non-invasive cardiac imaging, as previously described [19].

### Statistical analysis

Continuous variables are expressed as mean ± standard deviation if normally distributed and as median and interquartile range (IQR) if not normally distributed. Normality of the data was examined by histograms and the Shapiro–Wilk test. The CMR-derived independent variables (exposures) were LV end-diastolic and end-systolic volumes, LV mass, LVEF, detection and quantitation of LV myocardial fibrosis, and LV aneurysm. LV volumes were indexed to body surface area. LVEF was assessed continuously and based on a sensitivity analysis to determine the best cutoff point related to the highest risk prediction model performance. LV fibrosis was expressed as the total amount of myocardial fibrotic mass, percentage of LV mass, and categorically based on its presence or absence [13]. LV fibrosis mass was log-transformed because of skewed distribution. LV aneurysm was defined by the presence of wall thinning, motion abnormality (akinesia or dyskinesia), and late gadolinium enhancement. Cox proportional hazard models were used to verify the association of demographics, CMR-derived parameters, and traditional cardiovascular risk factors (hypertension, diabetes mellitus, dyslipidemia, and smoking) with the outcome. Parameters significantly related to the outcome in the univariate analysis were included in a multivariable model. Risk prediction model performance was evaluated by Harrell’s C statistic. Unadjusted Kaplan–Meier curves were performed to estimate survival distributions according to categorical exposures for the primary combined outcome. The log-rank test was used to compare survival curves. Pearson’s correlation coefficient was used to test the relationship between CMR-derived parameters. A p-value lower than 0.05 was considered statistically significant. All statistical analyses were performed using Stata (version 15.1, Stata Corporation, College Station, Texas, USA).

## Results

### Baseline characteristics

Baseline characteristics of the 113 patients included in the study are presented in Table 1. A total of 58 (51%) participants were women. Median age was 56 years (IQR: 45–67). In addition to cardiomyopathy, 13 patients also had evidence of the digestive form of Chagas disease, including dysphagia, megaesophagus, and megacolon. Patients were mainly at New York Heart Association (NYHA) functional class I (80%), followed by functional class II (15%) and functional class III (5%).

**Table 1:** Baseline characteristics of the participant patients

Demographics and clinical data	
Total amount of participants	113
Female	58 (51%)
Age (years)	56 [45–67]
BMI (Kg/m^2^)	26.4 ± 4.6
NYHA functional class	
I	90 (80%)
II	15 (13%)
III	8 (7%)
Hypertension	43 (38%)
Diabetes mellitus	5 (4%)
Dyslipidemia	27 (24%)
Smoking^a^	
Never	72 (64%)
Former	26 (23%)
Current	10 (9%)
Medications	
ACE inhibitors or ARBs	64 (57%)
Beta-blockers	41 (36%)
Spironolactone	16 (14%)
Furosemide	26 (23%)
Thiazides	21 (19%)
Amiodarone	21 (19%)
Statins	23 (20%)
Aspirin	21 (19%)
Electrocardiography	
1st or 2nd degree AV block	30 (27%)
Left bundle branch block	9 (8%)
Right bundle branch block	57 (50%)
Left anterior fascicular block	42 (37%)
Premature ventricular contractions	17 (15%)
Low QRS voltage	13 (12%)

*ACE* angiotensin-converting enzyme, *ARB* angiotensin receptor blockers, *AV* atrioventricular, *BMI* body mass index, *NYHA* New York Heart Association

^a^Smoking: five missing data

Hypertension was the most frequent comorbidity (38%), while dyslipidemia, diabetes, and current smoking were present in 24%, 9%, and 9% of the patients, respectively. Most participants (57%) were in use of angiotensin-converting enzyme inhibitors or angiotensin receptor blockers, while beta-blockers and amiodarone were in use by 36% and 19%, respectively. Right bundle branch block was the most common ECG abnormality (50%).

CMR-derived LV parameters are summarized in Table 2. The median LVEF was 53% (IQR: 41–62). LV aneurysm was detected in 38 (34%) patients, mostly at the apex (n = 35) but also in the inferolateral wall (n = 3). As assessed by LGE, LV fibrosis was identified in 76 (67%) individuals. LV fibrosis mass quantification was not feasible in 4 patients due to a small LV scar.

**Table 2:** Cardiac magnetic resonance evaluation of the participants

LV end-diastolic volume (mL)	153 [125–216]
LV end-diastolic volume index (mL/m^2^)	87 [75–126]
LV end-systolic volume (mL)	70 [47–125]
LV end-systolic volume (mL/m^2^)	42 [29–73]
LV mass (g)	108 [89–138]
LV mass index (g/m^2^)	63 [53–78]
LVEF (%)	53 [41–62]
Reduced LVEF (< 40%)	25 (22%)
Presence of LV LGE, n (%)	76 (67%)
LV LGE mass (g)	6 [0–13]
LV LGE mass (%)	5 [0–10]
Presence of LV aneurysm, n (%)	38 (34%)
Apical	35 (31%)
Inferolateral	3 (3%)
RV end-diastolic volume (mL)	114 [93–132]
RV end-diastolic volume index (mL/m^2^)	46 [33–63]

*LVEF* left ventricular ejection fraction, *LGE* late gadolinium enhancement, *LV* left ventricular, *RV* right ventricular

### Follow-up results

During a median follow-up time of 6.9 years (IQR: 6.1–11.0), 29 (26%) patients presented with the primary outcome: 11 had cerebrovascular events (nine had an ischemic stroke, while two had a TIA), 16 had AF or atrial flutter (n = 14 and 2, respectively), and two had LV apical thrombosis identified by echocardiography (n = 1) and invasive ventriculography (n = 1). Another patient had an LV thrombus at the apex identified by the baseline CMR and, hence, was not accounted for in the time-to-event analysis.

A detailed description of the cerebrovascular events is provided in Table 3. Eight patients with cerebrovascular events had no significant cranial artery disease on imaging exams. Another patient who reached the primary outcome underwent cranial arteries imaging in an external service, with no report of significant abnormalities. Of the other two patients with cerebrovascular events, one had no cranial arteries imaging but presented with AF simultaneously with the diagnosis of stroke. The other patient had no cranial arteries imaging and no AF but had a low 10-year atherosclerotic cardiovascular disease risk at the event time, as estimated by the ASCVD risk estimator [20].

**Table 3:** Clinical characteristics at the time of the cerebrovascular event

P. Nº	Neurologic signs	Computed tomography	ECG	Echocardiography				Imaging of intra- and extracranial arteries
				LVEF	LV apex		Thrombus	
					Wall motion	Aneurysm		
2	Left hemiparesis	Ischemic stroke with hemorrhagic transformation	SR	28%	Global hypokinesia	Small	No	CTA with no significant stenosis
6	Transient right upper limb paresis	No signs of stroke	AF	26%	Global akinesia	No	No	Not available
62	Left hemiparesis and dysarthria	Ischemic stroke	SR	33%	Global hypokinesia	Small	Yes; LV apex	CTA with no significant stenosis
65	Transient left hemiparesis and dysarthria	Ischemic stroke	AF	35%	Global hypokinesia	No	No	CTA with no significant stenosis
75	Left hemiparesis and dysarthria	Ischemic stroke with hemorrhagic transformation	SR	< 40%^a^	Global dyskinesia	Large	Yes; LV apex	CTA with no significant stenosis
76	External report of focal neurologic deficit	Ischemic stroke	SR	36%	Regional dyskinesia	Small	No	CTA performed in an external service
95	Persistent right hemiparesis and dysarthria	Ischemic stroke	SR	46%	Global hypokinesia	No	No	MRA with no significant stenosis
96	Aphasia	Ischemic stroke	AR	17%	Global akinesia	No	No	MRA with no significant stenosis
101	Aphasia and left upper limb paresis	Ischemic stroke	SR	58%	No	No	No	Not available
104	Aphasia and right upper limb	No signs of stroke	SR	50%	Regional dyskinesia	Small	Yes; Left atrial appendage	CTA with no significant stenosis
105	Left hemiparesis	Ischemic stroke	SR	35%	Global hypokinesia	Small	No	Carotid US with no significant stenosis

*AF* atrial fibrillation, *CTA* computed tomography angiography, *ECG* electrocardiogram, *LV* left ventricular, *LVEF* left ventricular ejection fraction, *MRA* magnetic resonance angiography, *P. Nº* patient number, *SR* sinus rhythm, *US* ultrasound

^a^Subjective analysis due to poor echocardiographic acoustic windows

**Fig. 1:**
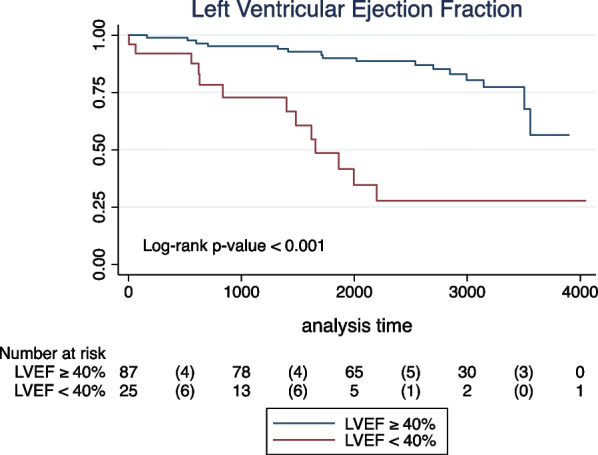
Kaplan–Meier unadjusted estimates for the primary combined endpoint (cerebrovascular event, atrial fibrillation/flutter, or intracavitary thrombus) in patients with Chagas cardiomyopathy according to left ventricular ejection fraction (LVEF) categories

**Fig. 2:**
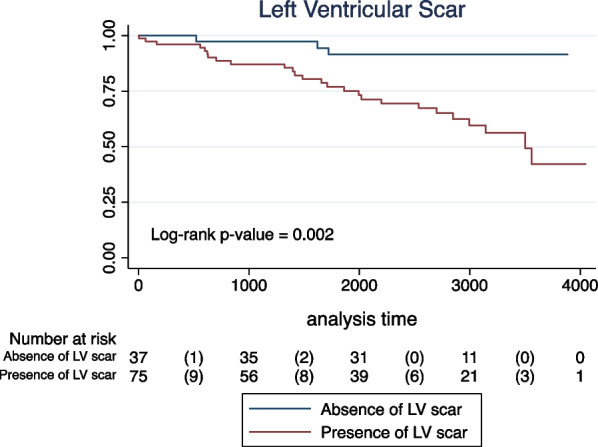
Kaplan–Meier unadjusted estimates for the primary combined endpoint (cerebrovascular event, atrial fibrillation/flutter, or intracavitary thrombus) in patients with Chagas cardiomyopathy according to the presence or absence of left ventricular (LV) scar

**Fig. 3:**
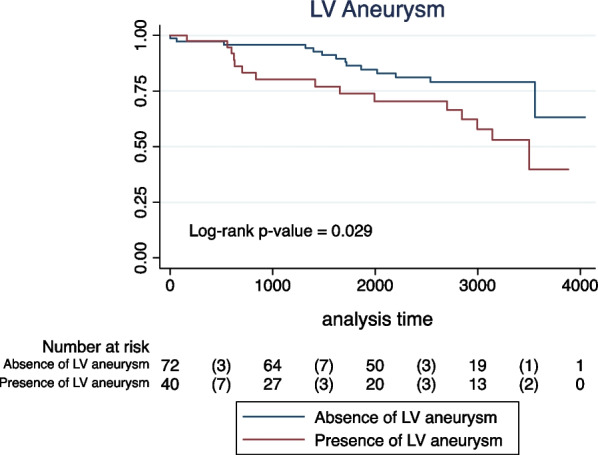
Kaplan–Meier unadjusted estimates for the primary combined endpoint (cerebrovascular event, atrial fibrillation/flutter, or intracavitary thrombus) in patients with Chagas cardiomyopathy according to the presence or absence of left ventricular (LV) aneurysm

In the univariate analyses, age and all CMR-derived parameters were significantly associated with the primary outcome (Table 4). Sex, hypertension, dyslipidemia, and smoking were not significantly to the combined endpoint. None of the patients with diabetes reached an event of interest, preventing Cox regression estimates. LVEF lower than 40% had the highest risk prediction performance among the LVEF cutoffs tested (C-statistic: 0.693), Additional file 2: Table S2. A total of 25 patients (22%) had a LVEF lower than 40%. Survival distributions according to LVEF categories, presence of LV scar, and LV aneurysm are demonstrated by Kaplan–Meier curves (log-rank p-value < 0.001, 0.002, and 0.029, respectively; Figs. 1, 2, 3).

**Table 4:** Univariate Cox proportional hazard models for the primary combined outcome (stroke, TIA, atrial fibrillation, atrial flutter, and intracavitary thrombus) in patients with chronic Chagas cardiomyopathy

	Univariate analysis		
	HR	95% CI	p-value
Demographic and clinical data^1^			
Age (years)	1.03	1.00–1.06	0.022
Sex (female vs. male)	0.74	0.35–1.53	0.419
Hypertension	0.97	0.46–2.07	0.955
Dyslipidemia	1.54	0.67–3.50	0.302
Smoking			
Never	Reference	Reference	Reference
Former	1.63	0.72–3.66	0.234
Current	0.48	0.06–3.61	0.476
CMR-derived parameters			
LV end-diastolic volume index (mL/m^2^)	1.01	1.00–1.01	< 0.001
LV end-systolic volume index (mL/m^2^)	1.01	1.00–1.01	< 0.001
LV mass index (g/m^2^)	1.02	1.01–1.04	< 0.001
LVEF (%)	0.94	0.92–0.96	< 0.001
LVEF < 40%	5.65	2.64–12.1	< 0.001
LV LGE (presence vs. absence)	5.31	1.60–17.61	0.006
LV LGE mass (g)^2^	1.73	1.23–2.43	0.001
LV LGE percentual mass^2^	1.70	1.18–2.44	0.004
LV aneurysm (presence vs. absence)	2.20	1.06–4.59	0.034

*CI* confidence interval, *LVEF* left ventricular ejection fraction, *HR* hazard ratio, *LGE* late gadolinium enhancement, *LV* left ventricular

^1^None of the patients with diabetes reached an event of interest, preventing Cox regression estimates

^2^Log-transformed variables

The multivariable models (Table 5) included age, LV mass, LVEF, LV scar, and LV aneurysm. LV end-diastolic and end-systolic volumes were not included in the adjusted model due to the higher correlation between them (r = 0.97, p < 0.001), and with LVEF (r = − 0.72 and − 0.82, respectively; p < 0.001 for both), Additional file 3: Table S3. The adjusted model 1 included LVEF and LV scar as continuous variables. Model 2 categorized LVEF as reduced (< 40%) or preserved (≥ 40%), while LV scar was assessed as to its presence versus absence. In the adjusted model 1, only a lower LVEF was significantly related to the primary combined outcome (HR: 0.96, 95% CI: 0.93–0.99, p = 0.014), C-statistic = 0.775. In the adjusted model 2, a reduced LVEF < 40% was significantly associated with the primary combined outcome (HR: 3.16, 95% CI: 1.38–7.25, p = 0.006), C-statistic: 0.806.

**Table 5:** Multivariable Cox proportional hazard models for the primary combined outcome (stroke, TIA, atrial fibrillation, atrial flutter, and intracavitary thrombus) in patients with chronic Chagas cardiomyopathy

	Multivariable analysis		
	HR	95% CI	p-value
**Model 1**			
Age (years)	1.02	0.99–1.06	0.144
LV mass index (g/m^2^)	1.00	0.98–1.02	0.373
LVEF (%)	0.96	0.93–0.99	0.014
LV LGE mass (g)	1.14	0.74–1.74	0.248
LV aneurysm (presence vs. absence)	1.74	0.71–4.23	0.221
**Model 2**			
Age (years)	1.02	0.99–1.06	0.089
LV mass index (g/m^2^)	1.01	0.99–1.02	0.215
LVEF < 40%	3.16	1.38–7.25	0.006
LV LGE (presence vs. absence)	2.46	0.66–9.18	0.179
LV aneurysm (presence vs. absence)	1.74	0.91–4.33	0.221

Model 1: Left ventricular ejection fraction and late gadolinium enhancement assessed as continuous variables

Model 2: Left ventricular ejection fraction and late gadolinium enhancement assessed as categorical variables

*CI* confidence interval, *LVEF* left ventricular ejection fraction, *HR* hazard ratio, *LGE* late gadolinium enhancement, *LV* left ventricular

Since the multivariable model included five parameters, we considered possible overfitting. Given that the primary question is to determine which of the CMR parameters is the most important predictor, we performed a sensitivity analysis including only LVEF, LV scar (both continuous variables), and LV aneurysms in a multivariable model. LV mass was not included in the model due to the correlation between LV mass and LVEF and because LV mass seemed relatively weaker on univariate analysis. In this additional model, LVEF was significantly related to the outcomes (HR: 0.95, 95% CI: 0.92–0.98, p = 0.001), but not either LV scar (HR: 1.25, 95% CI: 0.85–1.84, p = 0.247) or LV aneurysms (HR: 1.42, 95% CI: 0.62–3.26, p = 0.401).

The overall incidence rate of the combined event in our cohort was 4.2 new cases per 100 persons-years. In the group with reduced LVEF, the incidence rate for those events was 13.9 new cases per 100 persons-year, significantly different from those with preserved LVEF (incidence-rate of 2.6 new cases per 100 persons-year, p < 0.001). The incidence rate of cerebrovascular events in patients with reduced LVEF was 7.5 new cases per 100 persons-year.

## Discussion

This study demonstrates that LV systolic dysfunction is a significant predictor of cerebrovascular events and potential sources of thromboembolism in patients with Chagas cardiomyopathy. Although LV fibrosis and LV aneurysms have been associated with the combined outcome in the univariate analysis, this relationship was not significant in the adjusted models. Notably, our cohort’s overall incidence rate of stroke or TIA was relatively low. In addition, during the regular clinical follow-up, a higher proportion of potential sources of thromboembolic events, such as AF or flutter and LV mural thrombus, were identified, warranting preventive oral anticoagulation. Although thromboembolism is a well-recognized phenomenon in individuals with Chagas disease, data about risk factors for cerebrovascular events in this clinical condition are scarce, mostly from autopsy and clinical case series or case–control investigations [9, 21]. Hence, the results from our cohort are significant in several aspects.

First, using CMR, a reference method to assess LV structure and function, we found that reduced LVEF was significantly related to incident cerebrovascular events and potential sources of thromboembolism in patients with Chagas disease. This result is consistent with prior investigations using echocardiography to assess LV function. Sousa et al. found that LV systolic dysfunction was associated with cardioembolic ischemic stroke in a large cohort of patients with Chagas disease [22]. Nunes et al., in a study of patients with Chagas disease and impaired LV systolic function, also demonstrated that LVEF < 35% was related to ischemic cerebrovascular events [23]. More recently, Saraiva et al. showed that lower LVEF was a predictor of new-onset AF, although this relationship lost significance after adjustment for left atrial parameters. Unlike that study, which included patients predominantly in the indeterminate form of Chagas disease or with the cardiac form with isolated electrocardiographic changes, our investigation focused on those with evidence of chronic Chagas cardiomyopathy when the predicted value of LV structure and function is potentially higher [24].

**Fig. 4:**
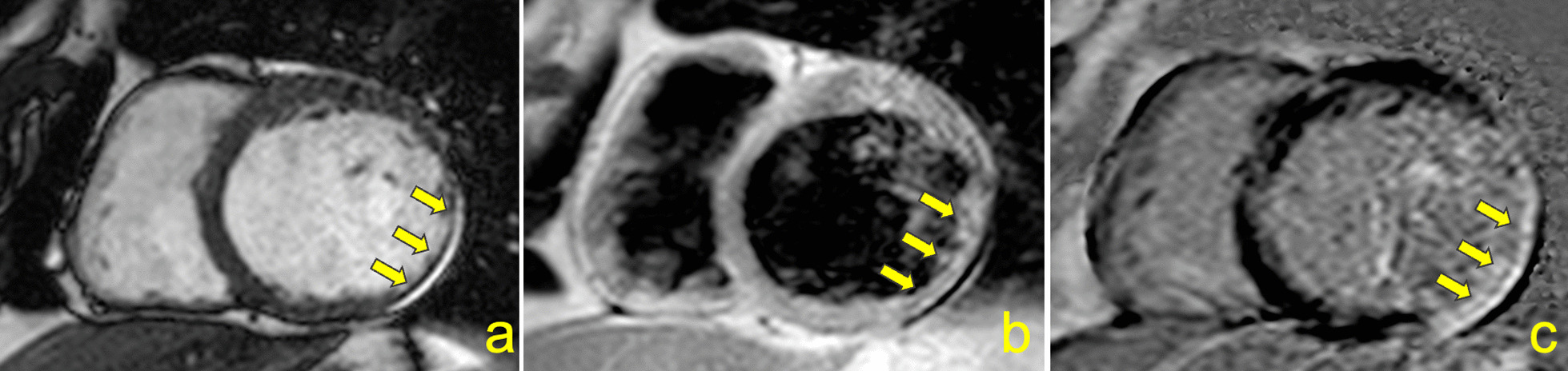
Cardiovascular magnetic resonance (CMR) of a 62 years-old male patient with chronic Chagas cardiomyopathy who developed ischemic stroke 1.7 years after the CMR examination. Left ventricular myocardial thinning is depicted in anterolateral and inferolateral segments from short axis cine-steady free precession (**a**) and black blood (**b**) pulse sequences, associated with late gadolinium enhancement (**c**). Baseline LVEF was 31%

Second, we tested the role of LV myocardial fibrosis in predicting cerebrovascular events and thromboembolic sources in Chagas disease (Fig. 4). Although CMR-derived LGE was associated with the combined outcome in the univariate analysis, this relationship lost significance in the adjusted model. However, because the number of events in our cohort was low, we recognize possible overfitting. Hence, further work is required to address whether LV myocardial fibrosis is an independent risk marker for stroke and sources of thromboembolic events in patients with Chagas disease.

Third, the presence of LV aneurysm was not related to the primary combined outcome in our study. In a sensitivity analysis, including only LV apical aneurysms, after excluding aneurysms of the inferolateral wall, the results did not significantly change. Although LV apical aneurysm is a well-known site of intramural thrombi formation, its role as an embolic event predictor in the absence of LV systolic dysfunction is less established. In a case–control analysis, Carod-Artal et al. showed that apical aneurysms were independently related to stroke in Chagas disease patients [6]. Similarly, in a retrospective cohort of patients with Chagas disease, Sousa et al. observed that LV apical aneurysm was an independent predictor of cardioembolic ischemic stroke [22]. In contrast with these two studies, and similarly to our present results, in a prospective cohort of individuals with Chagas cardiomyopathy, Nunes et al. found that LV apical aneurysm was not independently associated with ischemic cerebrovascular events [23].

**Fig. 5:**
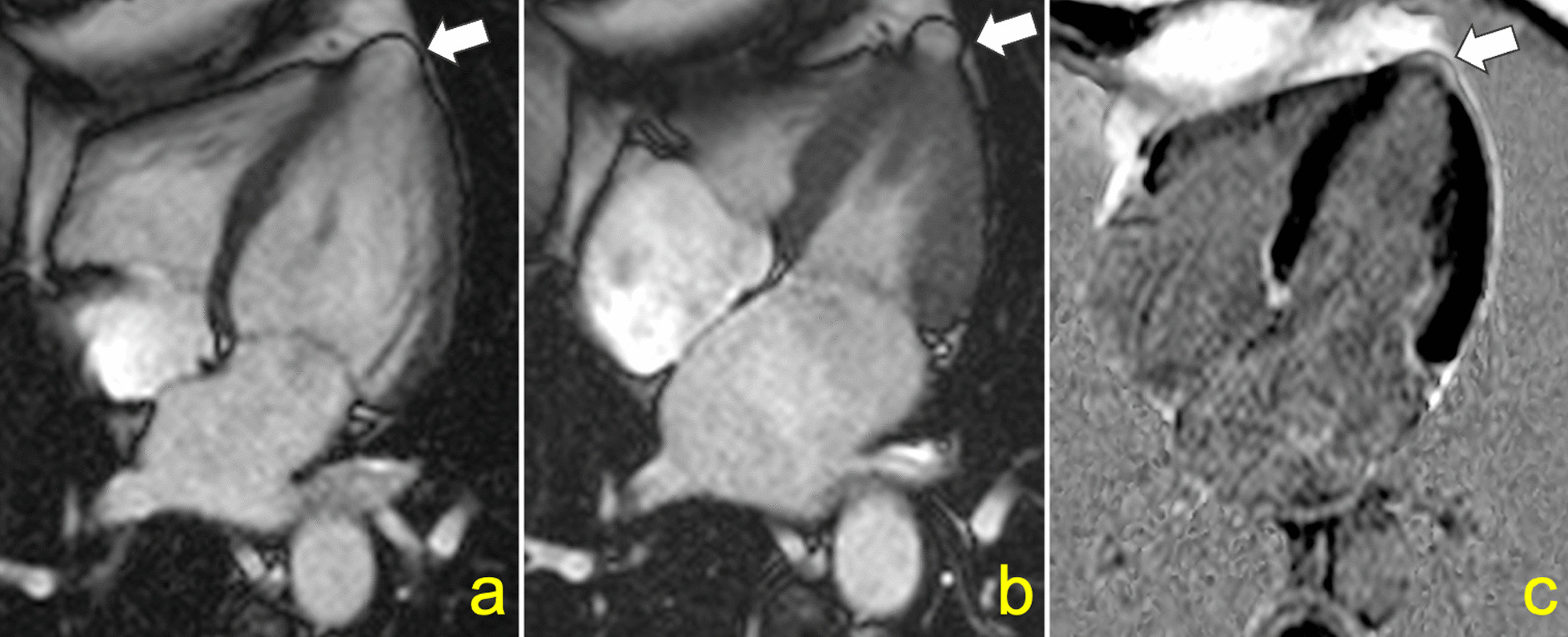
Small “hollow punch” left ventricular (LV) aneurysm in a 60 years-old female who developed atrial flutter 8.6 years after the CMR examination. A narrow neck aneurysm is depicted in diastole (**a**) and systole (**b**) in a four-chamber cine-steady state free precession pulse sequence. A small amount of late gadolinium enhancement at the LV apex can be seen (**c**). Baseline LVEF was 70%

**Fig. 6:**
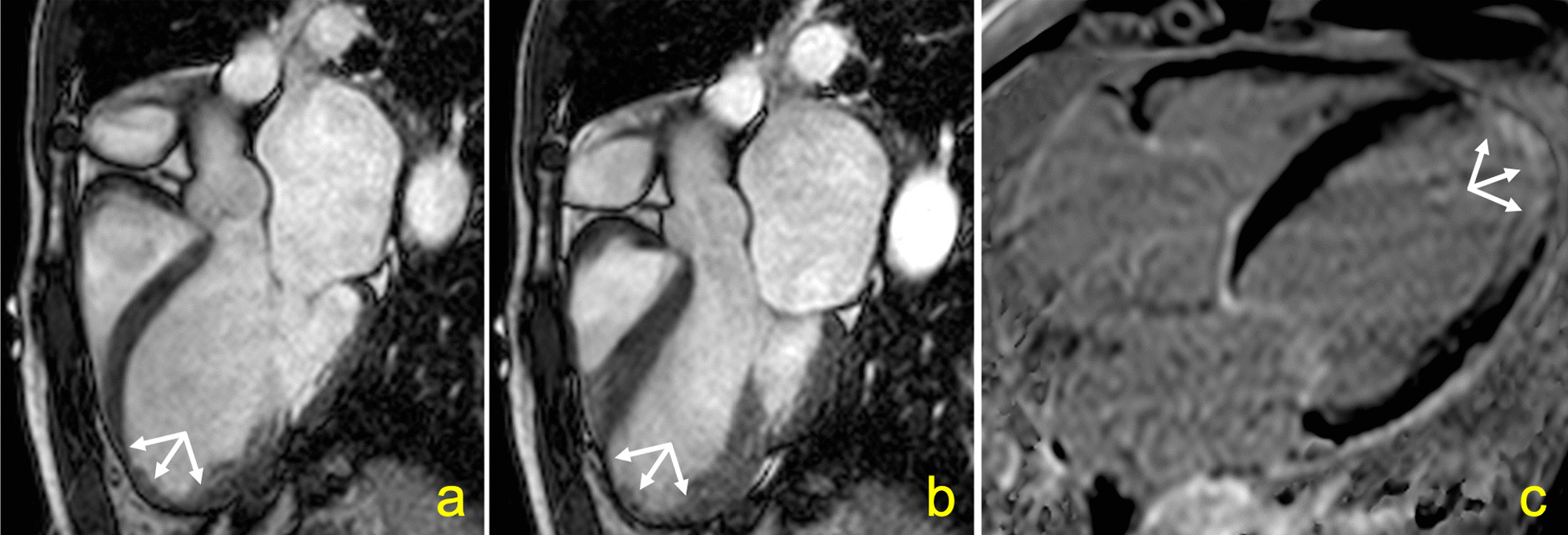
Large left ventricular aneurysm in a 67 years-old male who developed ischemic stroke 5.5 years after the CMR examination. A wide neck aneurysm is depicted in diastole (**a**) and systole (**b**) in a three-chamber cine-steady state free precession pulse sequence. A large amount of late gadolinium enhancement at the left ventricular apex can be seen (**c**). Baseline LVEF was 23%

In our view, these conflicting findings may result, at least in part, from the different methods and definitions used to describe LV aneurysms in those studies. Apical aneurysms in Chagas disease can range from small “hollow punch” lesions to large areas of akinetic myocardium thinning (Figs. 5 and 6). [25, 26] In our study, 26 (74%) of the LV apical aneurysms were classified as small lesions. Inadequate echocardiographic windows may limit the detection of the typical small LV apical aneurysms in Chagas disease patients, which can be present earlier in the disease course [27]. Of note, 8 out of 35 (23%) LV apical aneurysms identified in our study were found in patients with preserved LVEF and no other regional myocardial abnormality; all of these were small.

Finally, our study has described the incidence rate of cerebrovascular events and potential sources of thromboembolism in patients with Chagas disease and provided these data stratified by LV systolic dysfunction. The incidence of cardioembolic ischemic stroke in Chagas disease has been poorly described [28]. In another cross-sectional retrospective study of patients with cardiomyopathies, stroke cases were significantly more frequent in the Chagas group than in those with non-Chagas cardiomyopathies [29]. Overall, we found a relatively low incidence rate of either stroke or TIA in our cohort, with 1.6 new stroke cases per 100 persons-year. This finding could be explained by the heterogeneity of the study population, which included individuals with normal LV systolic function and hence low risk for cardioembolic events. Moreover, identifying a significant proportion of patients with incident sources of thromboembolism during the follow-up, especially AF and flutter, may also have played a central role in decreasing the stroke incidence rate in our cohort. These results highlight the importance of a specialized and regular follow-up focusing on monitoring risk factors for cardioembolic events, such as supraventricular arrhythmias and intracavitary thrombus [30]. In contrast to those with normal LV systolic function, patients with reduced LVEF showed a higher incidence rate of cerebrovascular events. This result agrees with another cohort of patients with Chagas cardiomyopathy and LV systolic dysfunction assessed by echocardiography, demonstrating an incidence rate of 2.7 ischemic cerebrovascular events per 100 patients-year [23]. In our cohort, those with LV systolic dysfunction showed an incidence rate of cerebrovascular events even higher as 7.5 new cases per 100 persons-year.

### Limitations

Given the retrospective nature of our study design, we acknowledge that causality cannot be inferred from our data. The diagnosis of stroke and TIA was primarily based on the medical reports. In one of the cases that reached the primary study outcome, we did not have access to the computed tomography images and results because the exam was performed in an external medical service. Nevertheless, we still could apply the World Health Organization criteria for stroke since this patient had persistent hemiparesis and dysarthria. One patient with a cerebrovascular event had normal LVEF and neither AF nor intracardiac thrombus at the time of the outcome, while images of cranial arteries were not available. Although this patient had a low 10-year atherosclerotic cardiovascular disease risk at the event time, we recognize that a non-cardioembolic cause for the cerebrovascular event could not be excluded. The other patients who had neither AF nor intracardiac thrombus at the time of the cerebrovascular event showed global LV dysfunction and no significant stenosis on cranial arteries imaging. For those, a cardioembolic cause is highly probable, although not definitive. A detached thrombus or paroxysmal AF may be a possible explanation for these events. Given the low-intensity, slowly progressive but incessant myocarditis in chronic Chagas cardiomyopathy, a serial evaluation with CMR would provide important insights into Chagas disease’s natural history [1]. In particular, CMR would offer a sensitive analysis of LV dysfunction progression and reveal the onset and expansion of LV fibrosis. Thus, a serial examination could re-stratify the patients during their lifespan. Unfortunately, we did not regularly repeat the CMR exams for this study. Of note, in a large cohort from the BENEFIT study, the authors showed a slow progression of cardiac changes in patients with Chagas disease in a follow-up of 5.4 years [31]. In our study, irregular pulse detected in clinical visits was considered a red flag for AF, triggering the request for an ECG or 24-h Holter monitoring. Prior studies have demonstrated that irregular pulse can be a quick and simple clinical sign to elicit AF, which a sensitivity of 95% and a specificity of 75% for this purpose [32]. Nevertheless, we acknowledge that the incidence of AF and flutter may be potentially underestimated, especially in the setting of paroxysmal events. Finally, we acknowledge that the incidence of the other outcomes may also be underestimated given the retrospective nature of our study.

## Conclusions

LV systolic dysfunction is a significant and independent predictor of cerebrovascular events and potential sources of thromboembolism in patients with chronic Chagas cardiomyopathy. The predictive value of LV fibrosis and aneurysm for these events needs further clarification, particularly in those with normal LVEF. A regular follow-up of patients with Chagas disease, with attention to LV systolic dysfunction and other risk factors for cardioembolic events, such as AF and intracavitary thrombus, might effectively reduce cerebrovascular events in this clinical condition. Nevertheless, further studies are needed to ascertain the potential benefit of this more frequent surveillance for preventing thromboembolic events in patients with chronic Chagas cardiomyopathy.

## Supplementary Information

**Additional file 1.** CMR pulse sequence parameters.

**Additional file 2.** Harrell's C-statistic for the primary combined outcome according to different LVEF cutoff points.

**Additional file 3.** Pearson's correlation coefficient between CMR-derived parameters.

## Data Availability

The datasets used and/or analyzed during the current study are available from the corresponding author on reasonable request.

## Abbreviations

AF: Atrial fibrillation
CI: Confidence interval
CMR: Cardiovascular magnetic resonance
ECG: Electrocardiogram
HR: Hazard ratio
IQR: Interquartile range
LGE: Late gadolinium enhancement
LV: Left ventricle/left ventricular
LVEF: Left ventricular ejection fraction
NYHA: New York Heart Association
*T. cruzi*: *Trypanosoma cruzi*
TIA: Transient ischemic attack

## References

[1] Rassi A, Rassi A, Marin-Neto JA (2010). Chagas disease. Lancet.

[2] WHO. Integrating neglected tropical diseases into global health and development: fourth WHO report on neglected tropical diseases. Geneva: World Health Organization; 2017, 2017.

[3] Meymandi SK, Forsyth CJ, Soverow J, Hernandez S, Sanchez D, Montgomery SP (2017). Prevalence of Chagas Disease in the Latin American-born population of Los Angeles. Clin Infect Dis.

[4] Requena-Mendez A, Aldasoro E, de Lazzari E, Sicuri E, Brown M, Moore DA (2015). Prevalence of Chagas disease in Latin-American migrants living in Europe: a systematic review and meta-analysis. PLoS Negl Trop Dis.

[5] Marin-Neto JA, Cunha-Neto E, Maciel BC, Simoes MV (2007). Pathogenesis of chronic Chagas heart disease. Circulation.

[6] Carod-Artal FJ, Vargas AP, Horan TA, Nunes LG (2005). Chagasic cardiomyopathy is independently associated with ischemic stroke in Chagas disease. Stroke.

[7] Carod-Artal FJ, Vargas AP, Melo M, Horan TA (2003). American trypanosomiasis (Chagas’ disease): an unrecognised cause of stroke. J Neurol Neurosurg Psychiatry.

[8] Montanaro VVA, Hora TF, da Silva CM, de Viana Santos CV, Lima MIR, de Jesus Oliveira EM (2019). Cerebral infarct topography of atrial fibrillation and Chagas disease. J Neurol Sci.

[9] Cardoso RN, Macedo FY, Garcia MN, Garcia DC, Benjo AM, Aguilar D (2014). Chagas cardiomyopathy is associated with higher incidence of stroke: a meta-analysis of observational studies. J Card Fail.

[10] Dias Junior JO, da Costa Rocha MO, de Souza AC, Kreuser LJ, de Souza Dias LA, Tan TC (2014). Assessment of the source of ischemic cerebrovascular events in patients with Chagas disease. Int J Cardiol.

[11] Carod-Artal FJ, Vargas AP, Falcao T (2011). Stroke in asymptomatic Trypanosoma cruzi-infected patients. Cerebrovasc Dis.

[12] Montanaro VV, da Silva CM, de Viana Santos CV, Lima MI, Negrao EM, de Freitas GR (2016). Ischemic stroke classification and risk of embolism in patients with Chagas disease. J Neurol.

[13] Volpe GJ, Moreira HT, Trad HS, Wu KC, Braggion-Santos MF, Santos MK (2018). Left ventricular scar and prognosis in chronic Chagas cardiomyopathy. J Am Coll Cardiol.

[14] Andrade JP, Marin-Neto JA, Paola AA, Vilas-Boas F, Oliveira GM, Bacal F (2011). I Latin American guidelines for the diagnosis and treatment of Chagas cardiomyopathy. Arq Bras Cardiol.

[15] Foulkes MA, Wolf PA, Price TR, Mohr JP, Hier DB (1988). The Stroke Data Bank: design, methods, and baseline characteristics. Stroke.

[16] World Health Organization. WHO STEPS Stroke Manual: the WHO STEPwise approach to stroke surveillance. World Health Organization, Noncommunicable Diseases and Mental Health. 2006:1–96.

[17] Hindricks G, Potpara T, Dagres N, Arbelo E, Bax JJ, Blomstrom-Lundqvist C (2021). 2020 ESC Guidelines for the diagnosis and management of atrial fibrillation developed in collaboration with the European Association for Cardio-Thoracic Surgery (EACTS): the Task Force for the diagnosis and management of atrial fibrillation of the European Society of Cardiology (ESC) Developed with the special contribution of the European Heart Rhythm Association (EHRA) of the ESC. Eur Heart J.

[18] Saoudi N, Cosio F, Waldo A, Chen SA, Iesaka Y, Lesh M (2001). A classification of atrial flutter and regular atrial tachycardia according to electrophysiological mechanisms and anatomical bases; a Statement from a Joint Expert Group from The Working Group of Arrhythmias of the European Society of Cardiology and the North American Society of Pacing and Electrophysiology. Eur Heart J.

[19] Massussi M, Scotti A, Lip GYH, Proietti R (2021). Left ventricular thrombosis: new perspectives on an old problem. Eur Heart J Cardiovasc Pharmacother.

[20] Lloyd-Jones DM, Huffman MD, Karmali KN, Sanghavi DM, Wright JS, Pelser C (2017). Estimating longitudinal risks and benefits from cardiovascular preventive therapies among medicare patients: the million hearts longitudinal ASCVD risk assessment tool: a special report from the American Heart Association and American College of Cardiology. J Am Coll Cardiol.

[21] Carod-Artal FJ, Gascon J (2010). Chagas disease and stroke. Lancet Neurol.

[22] Sousa AS, Xavier SS, Freitas GR, Hasslocher-Moreno A (2008). Prevention strategies of cardioembolic ischemic stroke in Chagas’ disease. Arq Bras Cardiol.

[23] Nunes MC, Barbosa MM, Ribeiro AL, Barbosa FB, Rocha MO (2009). Ischemic cerebrovascular events in patients with Chagas cardiomyopathy: a prospective follow-up study. J Neurol Sci.

[24] Saraiva RM, Pacheco NP, Pereira T, Costa AR, Holanda MT, Sangenis LHC (2020). Left atrial structure and function predictors of new-onset atrial fibrillation in patients with Chagas disease. J Am Soc Echocardiogr.

[25] Oliveira JS, Mello De Oliveira JA, Frederigue U, Lima Filho EC (1981). Apical aneurysm of Chagas’s heart disease. Br Heart J.

[26] Nunes MCP, Beaton A, Acquatella H, Bern C, Bolger AF, Echeverria LE (2018). Chagas cardiomyopathy: an update of current clinical knowledge and management: a scientific statement from the American Heart Association. Circulation.

[27] Acquatella H, Asch FM, Barbosa MM, Barros M, Bern C, Cavalcante JL (2018). Recommendations for multimodality cardiac imaging in patients with Chagas disease: a report from the American Society of Echocardiography in Collaboration With the Inter American Association of Echocardiography (ECOSIAC) and the Cardiovascular Imaging Department of the Brazilian Society of Cardiology (DIC-SBC). J Am Soc Echocardiogr.

[28] Mendes F, Mediano MFF, Silva RS, Xavier SS, do Brasil P, Saraiva RM, et al. Discussing the score of cardioembolic ischemic stroke in Chagas disease. Trop Med Infect Dis. 2020;5(2). 10.3390/tropicalmed5020082 PMC7345975 32466425

[29] da Matta JA, Aras R, de Macedo CR, da Cruz CG, Netto EM (2012). Stroke correlates in chagasic and non-chagasic cardiomyopathies. PLoS ONE.

[30] Perez-Molina JA, Perez AM, Norman FF, Monge-Maillo B, Lopez-Velez R (2015). Old and new challenges in Chagas disease. Lancet Infect Dis.

[31] Schmidt A, Dias Romano MM, Marin-Neto JA, Rao-Melacini P, Rassi A, Mattos A (2018). Effects of trypanocidal treatment on echocardiographic parameters in chagas cardiomyopathy and prognostic value of wall motion score index: a BENEFIT trial echocardiographic substudy. J Am Soc Echocardiogr.

[32] Dewar RI, Lip GY (2007). Identification, diagnosis and assessment of atrial fibrillation. Heart.

