# Supplementary material for: Association of left ventricular abnormalities with incident cerebrovascular events and sources of thromboembolism in patients with chronic Chagas cardiomyopathy
Source: J Cardiovasc Magn Reson. 2022 Nov 3;24:52. doi: 10.1186/s12968-022-00885-x (PMC9632087; doi:10.1186/s12968-022-00885-x)
Supplement: Supplementary file 1 — Additional file 1. CMR pulse sequence parameters. [file 12968_2022_885_MOESM1_ESM.docx]

| **Supplemental Table 1.** Cardiovascular magnetic resonance pulse sequence parameters. | | |
| --- | --- | --- |
|  | **Balanced Steady-State Free Precession** | **Inversion Recovery Fast Gradient-Echo** |
| **Repetition time (ms)** | 3.8 | 5.4 |
| **Echo time (ms)** | 1.6 | 1.3 |
| **Flip angle (degrees)** | 45 | 20 |
| **Number of acquisition phases** | 30 | 1 |
| **Matrix** | 256 x 160 | 256 x 192 |
| **Field of view (mm)** | 360-400 | 360-400 |
| **Slice thickness (mm)** | 8 | 10 |
| **Gap between slices (mm)** | 2 | No gap |
| **Inversion time (ms)** | - | 150-280 |
