# Supplementary material for: Association of left ventricular abnormalities with incident cerebrovascular events and sources of thromboembolism in patients with chronic Chagas cardiomyopathy
Source: J Cardiovasc Magn Reson. 2022 Nov 3;24:52. doi: 10.1186/s12968-022-00885-x (PMC9632087; doi:10.1186/s12968-022-00885-x)
Supplement: Supplementary file 2 — Additional file 2. Harrell's C-statistic for the primary combined outcome according to different LVEF cutoff points. [file 12968_2022_885_MOESM2_ESM.docx]

| **Supplemental Table 2.** Harrell’s C-statistic for the primary combined outcome according to different LVEF cutoff points. | |
| --- | --- |
|  |  |
| **LVEF cutoff point** | **Harrell’s C-statistic** |
| < 55% | 0.637 |
| < 50% | 0.625 |
| < 45% | 0.649 |
| < 40% | 0.693 |
| < 35% | 0.667 |
| LVEF: left ventricular ejection fraction | |
