# Supplementary material for: Association of left ventricular abnormalities with incident cerebrovascular events and sources of thromboembolism in patients with chronic Chagas cardiomyopathy
Source: J Cardiovasc Magn Reson. 2022 Nov 3;24:52. doi: 10.1186/s12968-022-00885-x (PMC9632087; doi:10.1186/s12968-022-00885-x)
Supplement: Supplementary file 3 — Additional file 3. Pearson's correlation coefficient between CMR-derived parameters. [file 12968_2022_885_MOESM3_ESM.docx]

| **Supplemental Table 3.** Person’s correlation coefficient between CMR-derived parameters. | | | | |
| --- | --- | --- | --- | --- |
|  | LV EDVI | LV ESVI | LV mass index | LVEF |
| LV EDVI | - | - | - | - |
| LV ESVI | 0.97 | - | - | - |
| LV mass index | 0.67 | 0.64 | - | - |
| LVEF | -0.72 | -0.82 | -0.61 | - |
| LV LGE mass ^1^ | 0.49 | 0.48 | 0.48 | -0.50 |
| ^1^ Log-transformed variable.  EDVI: end-diastolic volume index; ESVI: end-systolic volume index; EF: ejection fraction; LGE: late gadolinium enhancement; LV: left ventricular.  p-value < 0.001 for all correlations tested. | | | | |
